# One Year Follow-Up Risk Assessment in SKH-1 Mice and Wounds Treated with an Argon Plasma Jet

**DOI:** 10.3390/ijms18040868

**Published:** 2017-04-19

**Authors:** Anke Schmidt, Thomas von Woedtke, Jan Stenzel, Tobias Lindner, Stefan Polei, Brigitte Vollmar, Sander Bekeschus

**Affiliations:** 1 Leibniz-Institute for Plasma Science and Technology (INP Greifswald), Departments of Plasma Life Science and ZIK Plasmatis, Felix-Hausdorff-Str. 2, 17489 Greifswald, Germany; woedtke@inp-greifswald.de (T.v.W.); sander.bekeschus@inp-greifswald.de (S.B.); 2 Department of Hygiene and Environmental Medicine, University Medicine Greifswald, 17475 Greifswald, Germany; 3 Core Facility Multimodal Small Animal Imaging, 18057 Rostock, Germany; jan.stenzel@med.uni-rostock.de (J.S.); tobias.lindner@med.uni-rostock.de (T.L.); stefan.polei@med.uni-rostock.de (S.P.); 4 Institute for Experimental Surgery, Rostock University Medical Center, Schillingallee 69a, 18057 Rostock, Germany; brigitte.vollmar@med.uni-rostock.de

**Keywords:** dermal full-thickness wounds, *kINPen* plasma jet, plasma medicine, reactive oxygen and nitrogen species, risk evaluation, SKH1 mouse model

## Abstract

Multiple evidence in animal models and in humans suggest a beneficial role of cold physical plasma in wound treatment. Yet, risk assessment studies are important to further foster therapeutic advancement and acceptance of cold plasma in clinics. Accordingly, we investigated the long-term side effects of repetitive plasma treatment over 14 consecutive days in a rodent full-thickness ear wound model. Subsequently, animals were housed for 350 days and sacrificed thereafter. In blood, systemic changes of the pro-inflammatory cytokines interleukin 1β and tumor necrosis factor α were absent. Similarly, tumor marker levels of α-fetoprotein and calcitonin remained unchanged. Using quantitative PCR, the expression levels of several cytokines and tumor markers in liver, lung, and skin were found to be similar in the control and treatment group as well. Likewise, histological and immunohistochemical analysis failed to detect abnormal morphological changes and the presence of tumor markers such as carcinoembryonic antigen, α-fetoprotein, or the neighbor of *Punc 11*. Absence of neoplastic lesions was confirmed by non-invasive imaging methods such as anatomical magnetic resonance imaging and positron emission tomography-computed tomography. Our results suggest that the beneficial effects of cold plasma in wound healing come without apparent side effects including tumor formation or chronic inflammation.

## 1. Introduction

Cold atmospheric pressure plasma has emerged as a promising tool for biomedical and clinical applications [1]. In this field called plasma medicine, encouraging results have been achieved for disinfection purposes [2], in vitro [3,4,5] and in patients [6,7,8]. Notably, a key feature determining healing is the state of wound oxygenation [9], and evidence suggests that the scavenging of active oxygen species impairs wound healing [10]. Accordingly, strategies influencing redox signaling may be used as an accessory therapy in chronic wound management [11,12,13], establishing a link to plasma medicine [14,15,16].

Cold physical plasmas are partially ionized gases that mediate biological responses especially via generation of reactive oxygen (ROS) and nitrogen species (RNS) [17,18,19]. Crucially, such species are translated in cells via redox enzymes [20] and therefore actively participate in intracellular signaling events [21,22,23]. Most mammalian cells maintain and benefit from a residual concentration of ROS and possess a complex system to sense a relay of ROS-related signals [24,25]. In contrast to low ROS and RNS quantities, higher concentrations are known to be responsible for apoptotic signaling [26,27,28] or finally for DNA damage [29]. Previous studies assessed the mutagenic risks of plasma in vitro [30,31,32]. The data provided in these studies demonstrated the absence of mutagenic or genotoxic effects in plasma-treated cells or in a hen’s egg test model for micronuclei induction (HET-MN), suggesting that a clinical application of the argon plasma jet does not pose mutagenic risks. This is confirmed by a clinical long-term observation of laser skin lesions which were treated by cold atmospheric plasma [33,34]. However, systematic in vivo studies investigating any malignant side effects of plasma have not carried out to date.

To detect tumorigenicity, the usage of rodent models is commonly proposed. With regard to potential plasma applications in human wound healing, we utilized a full-thickness immunocompetent mouse model subjected to plasma treatment to monitor long-term effects. Non-invasive methods such as magnetic resonance imaging (MRI) and positron-emission tomography/computed tomography (PET/CT) are able to detect neoplastic lesions throughout the body. Using both technologies, tumorigenic effects were investigated in animals one year after plasma treatment. Using quantitative PCR, ELISA measurements, and immunohistochemical analysis of several tumor markers, we also investigated primary tumor formation or metastasis of malignant tumors.

## 2. Results

### 2.1. Evaluation of Histological Architecture and Inflammation Status after One Year

**Figure 1:**
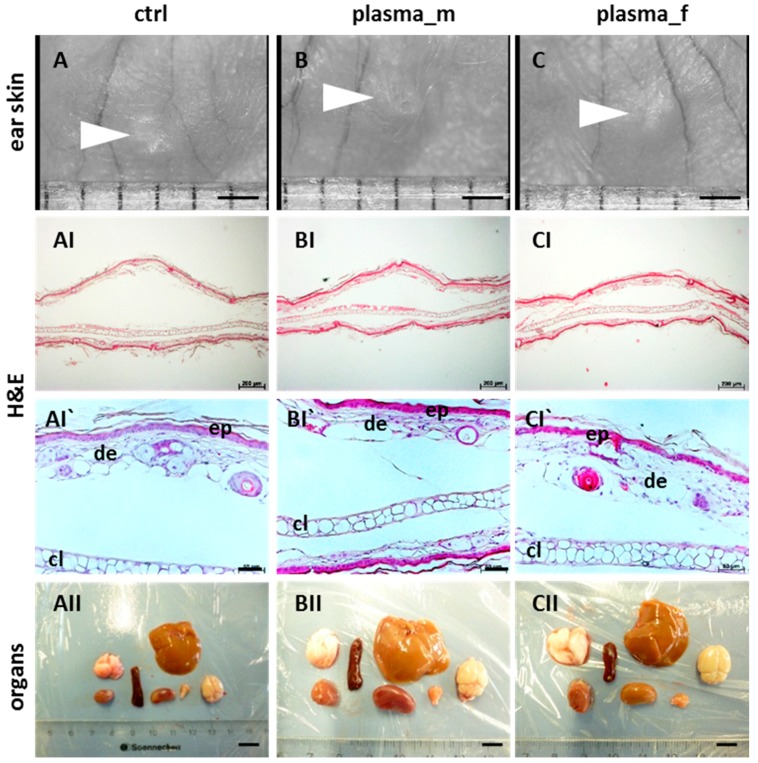
Macro- and microscopic skin wounds and organs 350 days after plasma treatment. Similar to untreated control animals (**A**), stereo microscopy of wound region revealed no differences of morphology (arrowheads) in ear tissue 350 days after injury in plasma- and untreated males (**B**) and females (**C**). Hematoxylin and eosin (H&E)-stained skin sections of dermal layers in control and plasma-treated mice were similar showing normal dermal architecture with a disconnection between the cartilage layer (cl) and dermis (de, ep, epidermis) at the wound site without inflammatory cell infiltration into the dermis (**I**–**I`**). Macroscopic evaluation of different organs (lung, spleen, liver, heart, kidney, thyroid glands, and brain) lacking visible tumor formation (**II**). Representative images are shown. Scale bar 1 cm (**II**), 1 mm (**A**–**C**), 100 µm (**I**), and 50 µM (**I`**).

The aim of this study was the risk evaluation of cold plasma 350 days after wound treatment in a total of 84 hairless mice. In our long-term observation, plasma-treated mice showed typical health state, nutrition, and behavior. Moreover, we did not identify any toxic side effects or chronic wound infections. Two untreated animals developed a hepatocellular carcinoma (HCC, male) or skin abnormalities (female), which served as positive controls for ex vivo analyses. One of forty-two animals in the plasma group showed an enlargement of the organ spleen (data not shown). Structurally, wound areas were similar between experimental groups (arrowhead in Figure 1A–C). Yet, hematoxylin and eosin (H&E) staining showed some histologic changes in the dermal wound region. We found a separation and disconnection of dermal layers (I) without inflammatory cell infiltration into the dermis (I`). This effect was observed in most animals independent from treatment regime or gender (controls, A; males, B; females, C), indicating a normal healing output without excessive scar formation on day 350. Macroscopically, the organs of plasma-treated animals did not exhibit morphological changes, signs of tumor formation or metastatic processes, or differences in size or weight, such as in lung, liver, brain, thyroid gland, kidney, spleen, and heart (II, clockwise rotation).

**Figure 2:**
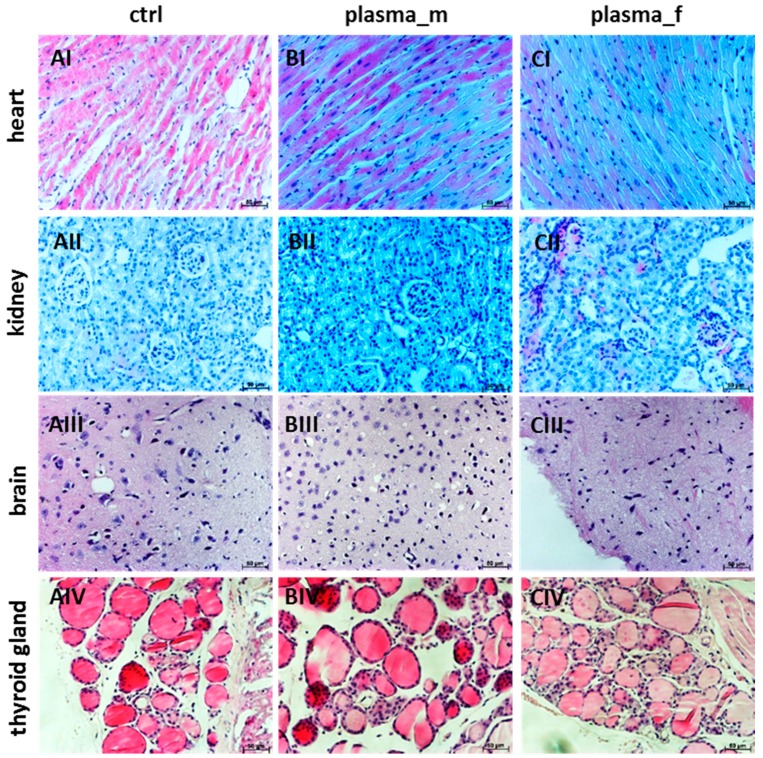
Histology in different organs of mice treated with plasma. H&E-stained heart sections showing normal architecture with cardiac myocytes and centrally placed nuclei (**I**) in controls (**A**), and plasma-treated males (**B**) and females (**C**). Kidney sections showing normal histological structure with glomerulus and convoluted tubules without necrosis (**II**). Brain sections from mice treated with plasma showing no signs of either neuronal injury or focal inflammatory cell infiltration nor hyperchromatic cells or cellular shrinkage or swelling (**III**). Normal thyroid histology showing follicles surrounded with thyrocytes without cellular infiltrations (**IV**). One representative picture of H&E staining is shown for selected organs. Scale bar 50 µm.

Next, H&E staining was performed for the heart, kidney, brain, and thyroid tissues (Figure 2). Normal architecture of the heart with cardiac myocytes and centrally placed nuclei (I) was observed. Similarly, kidney tissues showed typical glomeruli and convoluted tubule structures without signs of necrosis (II). Brain sections from mice treated with plasma did not reveal either neuronal injury, focal inflammatory cell infiltration, hyperchromatic cells, or cellular shrinkage or swelling (III). In thyroid histology, follicles were surrounded with thyrocytes without cellular infiltrations (IV).

**Figure 3:**
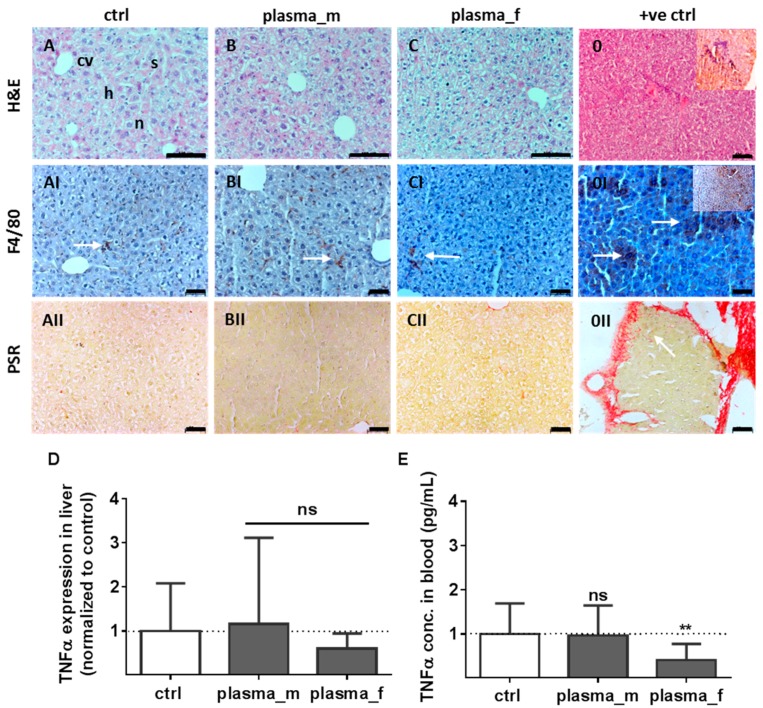
Liver histology, Kupffer cells, local and systemic tumor necrosis factor α (*TNFα*) levels. Similar to controls (**A**), H&E-stained liver sections from mice treated with plasma showing normal hepatic architecture with central vein (cv) and surrounding hepatocytes (h), sinusoids (s), and nuclei (n) in males (**B**) and females (**C**). Scale bar 100 µm. Immunostaining with F4/80 antibody was performed to show scattered inflammatory cell aggregations and to visualize ramified Kupffer cells in liver sections, which were not swollen or amoeboid (**AI**–**CI**). A hepatocellular carcinoma in liver (from the untreated control group) showed a strong cell infiltration (inlet, (**0**)) hyperplasia of liver tissue and a broad F4/80 positive cell-staining (inlet in (**0I**)) with inflammatory foci (arrows in (**0I**), +ve ctrl). Picrosirius red (PSR) staining of tissue slices from liver showing collagen fibers in cancer tissue of a hepatocellular carcinoma (HCC)-bearing mouse (arrow, (**0II**)) in contrast to non-tumor sections of plasma- and untreated animals (**A**–**CII**). Representative images are shown with scale bars of 50 µm (**I**–**II**). Using quantitative polymerase chain reaction (qPCR), *TNFα* mRNA expression was quantified in the liver of plasma- and untreated mice (**D**). Analysis of blood samples has shown a significant down-regulation of *TNFα* in females 15 days after wounding (**E**). Results are means + S.D. for *n* > 4 (**D**; ns, not significant) or nine mice (**E**, ** *p* < 0.01).

Similar to the controls (A), H&E-stained liver sections from mice treated with plasma showed normal hepatic architecture with the central vein (cv), radially surrounding hepatocytes (h), sinusoids (s), and nuclei (n) in males (B) and females (C). Additionally, we could not identify swollen or multinucleated macrophages in H&E staining, or scattered inflammatory cell aggregation (Figure 3A–C). Immunostaining with F4/80 antibody was performed to show the morphological architecture associated with a non-activated state. In an untreated control mouse we found a hepatocellular carcinoma (HCC) with a remarkable cellular infiltration in the liver (inlet in 0), which served as the “positive control” (+ve ctrl). The major histologic finding in the liver of the HCC-bearing mouse was a broad F4/80 positive cell staining, suggesting a strong inflammation with inflammatory foci (arrows, 0I). Contrary to that, we found in all mice studied a normal ramified structure of Kupffer cells. In addition, we observed no differences in the quality and amount of F4/80 positive cells, which were not swollen or amoeboid (AI–CI). One remarkable pathological characteristic of the liver is increased production of collagen, which is the main component of the extracellular matrix in fibrotic tissue [35]. Depositions of collagen fibers were examined in liver sections showing no hyperplasia of the fibrous tissue in plasma-treated mice (II). In contrast, arrows indicate a high level of collagen deposition stained by picrosirius red (PSR) in the fibrotic septa between nodules of HCC-bearing liver tissue (0II). Moreover, mRNA for the pro-inflammatory cytokine tumor-necrosis factor α (*TNFα*) was not elevated in the liver of plasma-treated mice relative to controls after one year (D). This finding was confirmed systemically in blood plasma, with a small but significant down-regulation of *TNFα* in females (E) 15 days after wounding (Figure 3).

**Figure 4:**
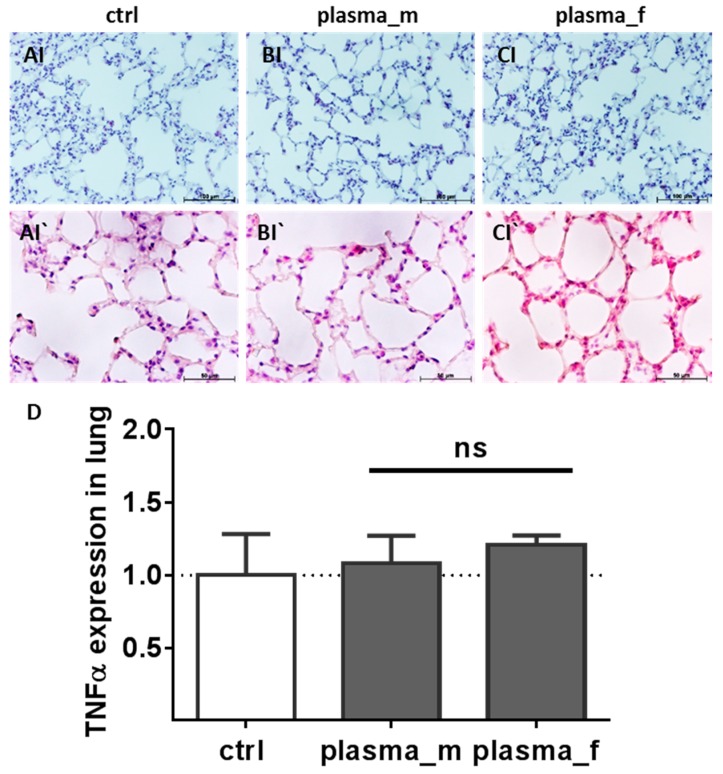
Lung histology and TNFα expression. Similar to controls (**A**), H&E staining did not show scattered inflammatory cell aggregations with interstitial lymphocytic and neutrophilic infiltration, thickening of the alveolar capillary membrane, or focal hemorrhage in the mesenchyme of lungs of plasma-treated males (**B**) or females (**C**). Using qPCR, *TNFα* mRNA expression was quantified in the lungs of plasma- and untreated mice (**D**). Representative images with 100 µm (**I**) or 50 µm (**I`**). Results are means + S.D. for *n* > 4 (**D**; ns, not significant).

Histological examination of the lung sections did not show changes consistent with lung pathology. Macroscopically, lungs were not less aerated or covered with white fibrin patches. Moreover, in H&E stained sections we did not find scattered inflammatory cell aggregations with interstitial neutrophilic infiltration, septal thickening of the alveolar capillary membrane, or focal hemorrhage of the mesenchyme in plasma-treated males (B) or females (C) similar to the controls (A). Likewise, *TNFα* mRNA expression was not altered in the lungs of plasma-treated mice relative to controls (Figure 4D).

**Figure 5:**
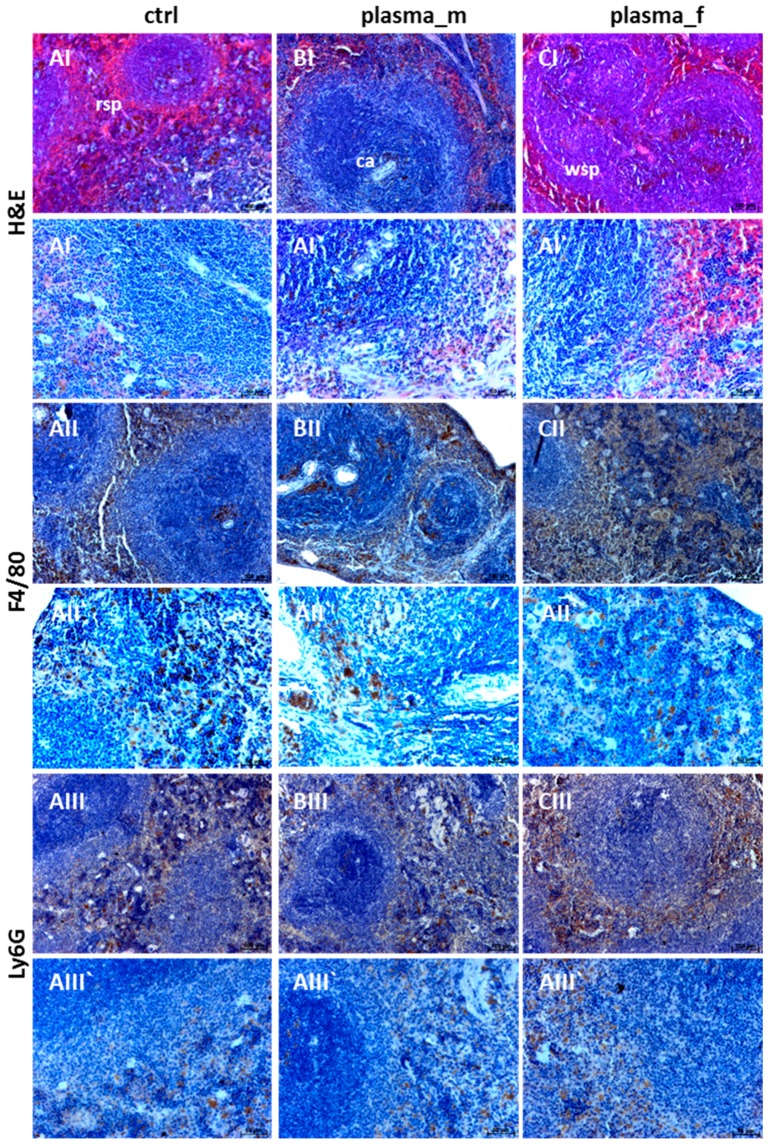
Macrophage and granulocyte distribution in spleens. Similar to controls (**A**), H&E staining did not show abnormalities in spleens in plasma-treated males (**B**) or females (**C**); rsp, red splenic pulp; ca, central artery; wsp, white splenic pulp. Intra-individual differences appeared in relation to the size of white and red pulp without a clear tendency. Immunostaining with F4/80 and Ly6G antibodies were performed to visualize macrophages (**II**) as well as normally dispersed granulocyte distribution (**III**). Representative images with 100 µm (**I**–**III**) or 50 µm (**I`**–**III`**).

Next, no morphological abnormalities of the spleen or excessive neutrophil infiltration in the red splenic pulp were found in plasma-treated mice (B,C) similar to untreated controls (A). Immunostaining with F4/80 antibody was performed to visualize macrophages (II) in the spleen and immunostaining with Ly6G showed the granulocytes distribution (III). Taken together, experimental groups exhibited no differences in staining of immune cell types (Figure 5).

**Figure 6:**
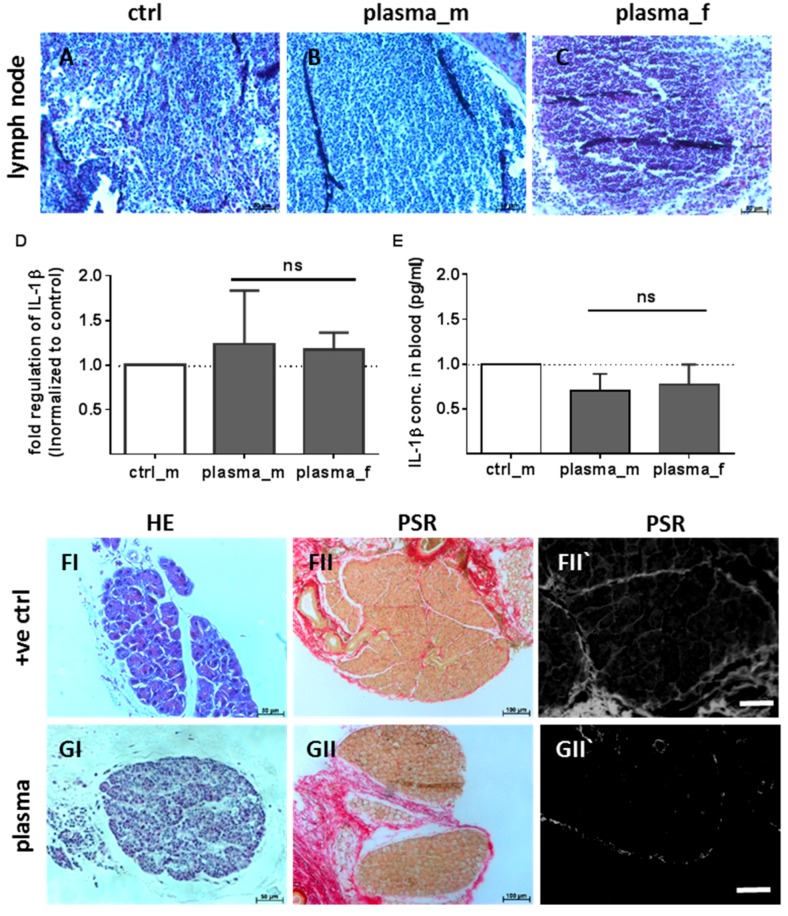
Lymph node histology, *IL-1β* expression, and comparison to tumor samples. H&E staining did not show hypertrophy, hyperplasia, or dilated parafollicular zones in lymph nodes of plasma-treated animals in comparison to controls (**A**) in males (**B**) and females (**C**). *IL-1β* mRNA expression was quantified by qPCR in cervical lymph nodes ((**D**), *n* > 4) and cytokine measurement of *IL-1β* concentration in blood samples 15 days after injury ((**E**), *n* > 9). Parafollicular hyperplasia, plasmocytosis, and increased collagen fiber density in lymph nodes of mice carrying a primary hepatocellular carcinoma (**F**) in contrast to plasma-treated mice (**G**) was visualized by H&E (**I**) and PSR staining (**II**) using light (**I**–**II**) or fluorescence microscopy (**II`**). Scale bar 50 µm (**A**–**C**), 100 µm (**F**,**G**).

Cervical lymph nodes are normally subject to a number of different pathological conditions including tumor development and inflammation [36]. Therefore, cervical lymph nodes were removed from plasma-treated animals and compared to untreated animals. The size of lymph nodes were similar in both experimental groups indicating the absence of lymphomas (data not shown). H&E staining showed neither follicular hyperplasia nor dilated parafollicular zones (Figure 6A–C). In the lymph nodes of plasma-treated mice, interleukin 1β (*IL-1β*) mRNA was in the range of control levels (Figure 6D). Moreover, blood plasma levels of *IL-1β* were similar in all treatment groups 15 days after wounding (Figure 6E). Additionally, parafollicular hyperplasia, plasmocytosis, and increased collagen density in the lymph nodes of several tumor models are reported [37]. We clearly obtained a follicular hyperplasia in the HCC-bearing mouse (Figure 6FI) in contrast to the plasma-treated mice (Figure 6GI). The collagen fiber density in cervical and mesenteric lymph nodes from this mouse was significantly increased compared to plasma and untreated mice, indicating no transformational changes in the latter groups. Collagen fibers are shown in red in a PSR staining using light microscopy (Figure 6FII) or by fluorescence microscopy (Figure 6GII) [38]. No histologic abnormalities of bones and other unmentioned organs were observed (data not shown).

### 2.2. Evaluation of Tumor Marker (TM) Expression and Secretion

**Figure 7:**
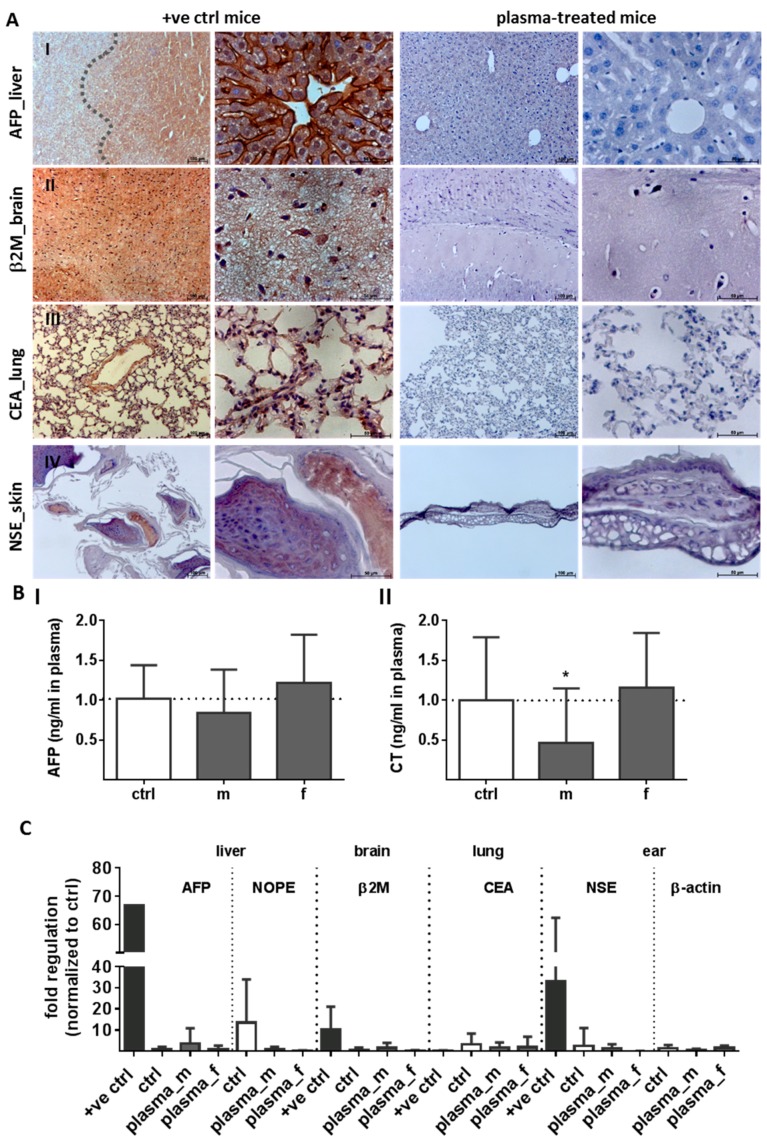
Unchanged tumor marker levels in liver, lung, brain, and skin of plasma-treated mice. Representative images of immunohistochemistry of different tumor markers (TM): *AFP* (**I**) in liver, *β2M* in brain (**II**), *CEA* in lung (**III**), as well as *NSE* staining in skin tissue (**IV**) after one year in positive control (+ve ctrl, left) and plasma-treated (right) animals (**A**). Scale bar 50 µm (right columns) or 100 µm (left columns). Using ELISA, we analyzed *AFP* (**I**), and calcitonin (*CT*) (**II**), a TM of medullary thyroid carcinoma, in blood serum (**B**; * *p* < 0.05; (*n* > 9). The mRNA expression levels of five TM and β-actin in liver, brain, lung, and ear skin tissues from plasma- and untreated (ctrl) mice were compared with organs from a HCC-bearing mouse (+ve ctrl). At least three independent experiments were performed and summarized in the indicated experimental groups (m, male; f, female). *AFP*, α-fetoprotein; *NOPE*, neighbor of *Punc 11*; *β2M*, β2-microglobulin; *NSE*, neuron specific enolase; *CEA*, carcinoembryonic antigen (**C**).

To explore the safety aspects of cold plasma as a precondition for a clinical application, we next investigated the tumor marker (TM) expression in several organs, skin tissue, and blood plasma using immunohistochemistry, quantitative polymerase chain reaction (qPCR), and ELISA 350 d after plasma treatment. Representative images of immunohistochemical staining revealed no enhanced tumor marker expression in liver, brain, lung, and skin tissue (Figure 7A). The immunohistochemical staining of α-fetoprotein (*AFP*, I), a traditional tumor marker for hepatocellular carcinoma (HCC), β2 microglobulin (*β2M*, II), a tumor marker for some blood cell cancers such as lymphoma and multiple myelomas [39], as well as the carcinoembryonic antigen (*CEA*, III), the most widely used tumor marker in patients with non-small cell lung cancer [40], were significantly enhanced in organs of the HCC-bearing mouse (+ve ctrl, left panel) compared to plasma-treated mice (right panel). Additionally, the neuron specific enolase (*NSE*), a TM of neuroendocrine tumors such as Merkel-cell carcinoma of the skin [41], was obviously increased in skin sections of the positive control mouse (+ve ctrl), which has developed a skin abnormality (IV, left). Nevertheless, no positive *NSE* staining was found in plasma-treated mice (IV, right). Blood plasma analysis of tumor markers revealed no differences between groups in *AFP* (I) or calcitonin level (*CT*, II), a TM of medullary thyroid carcinoma [42] (Figure 7B).

Next, we performed quantitative PCR analysis of five tumor markers such as *AFP*, *NOPE* (neighbor of *Punc 11*), a novel TM of the liver [43], β2 microglobulin (*β2M*), carcinoembryonic antigen (*CEA*), or neuron-specific enolase (*NSE*). No changes were observed in, e.g., liver, brain, lung, or ear skin tissue relative to the controls and in contrast to organs of the HCC-bearing mouse (+ve ctrl). Actin-containing elements of the cytoskeleton are changed in pathophysiological conditions such as malignant tumors [44]. We investigated alteration of either β-actin protein expression in skin tissue sections using IHC as well as gene expression using qPCR. Structural changes of the cytoskeleton and changes in mRNA levels were absent in plasma-treated mice (Figure 7C).

### 2.3. Multimodal Imaging of Mice 350 Days after Plasma Treatment

**Figure 8:**
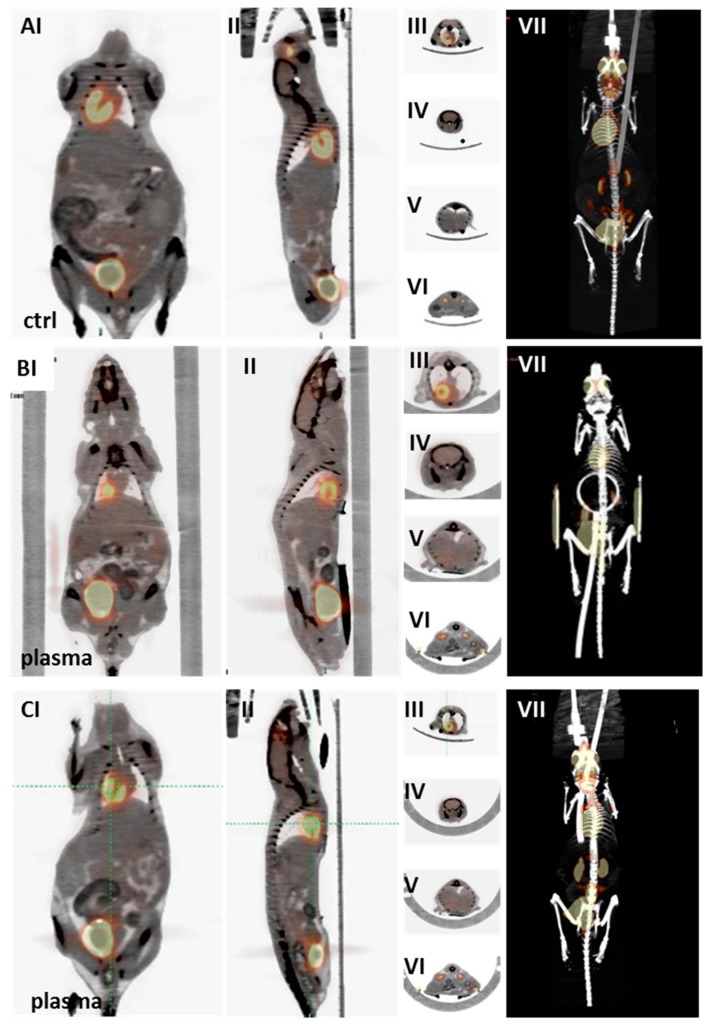
Positron emission tomography-computed tomography (PET/CT) imaging with 18F-fluorodeoxyglucose (18F-FDG) tracer for risk estimation after plasma treatment. (PET/CT with 18F-FDG tracer indicates tissue metabolic activity corresponding to glucose uptake. The spatial resolution was 1.5 mm. Representative scans of control (**A**), plasma-treated male (**B**), and female (**C**) mice after one year: whole body (**I**,**II**), heart (**III**), brain (**IV**), liver (**V**), and kidney (**VI**) scans and complete three-dimensional body PET/CT fusion image (**VII**).

**Figure 9:**
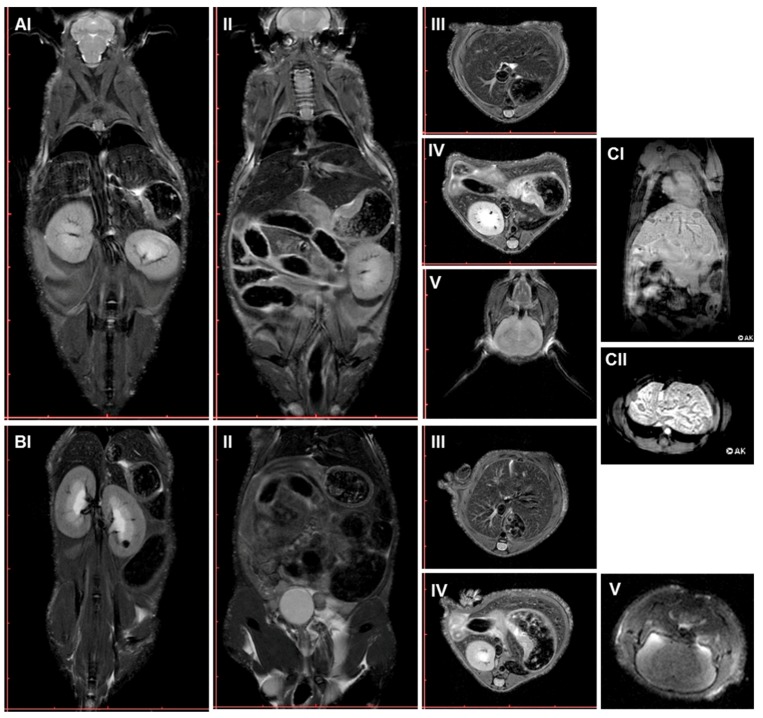
Risks evaluation in plasma-treated mice using anatomical magnetic-resonance imaging (MRI). Representative MRI scans of control (**A**) and plasma-treated mice one year after plasma treatment (**B**). Images derived from T2-weighted rapid acquisition with relaxation enhancement (RARE)-sequences with a deliverable spatial resolution of 0.18 mm in-plane, echo time (TE/TR 29/4100 ms): coronal slices with obvious visualization of kidneys (**I**) or stomach (**II**), as well as axial scans of abdomen with liver (**III**), kidney (**IV**), and head with the brain (**V**). Two representative images of a liver tumor are shown in a control (not plasma-treated) mouse (**CI**–**II**).

Positron-emission tomography/computed tomography (PET/CT) and anatomical magnetic resonance imaging (MRI) were employed to detect any long-term side effects in the plasma group. In PET/CT imaging with 18F-FDG, indicating tissue metabolic activity corresponding to glucose uptake, the plasma-treated male as well as female mice showed no significant differences in tracer uptake in the heart (III), brain (IV), liver (V), or kidney (VI). Throughout the body (VII), pathological differences in tracer uptake were absent in the organs (e.g., thymus) of plasma-treated and control mice (Figure 8). Similarly, representative three-dimensional MRI scans derived from T2-weighted rapid acquisition with relaxation enhancement (RARE)-sequences (at an in-plane resolution of 180 µm) did not show any indication of tumor formation in control (A) or plasma-treated mice (B) one year after plasma treatment (Figure 9). Longitudinal and axial whole body view did not show pathophysiological glucose uptake in the kidneys (I) or stomach (II). This was confirmed by lateral scans of the abdomen with liver (III), kidney (IV), and head with brain (V). By contrast, glucose uptake is shown in two representative images of a liver tumor in non-plasma-treated animals (CI–II).

## 3. Discussion

Chemical and physico-chemical clinical interventions require safety testing with regard to cytotoxicity and genotoxicity. Although cell culture experiments are commonly used to investigate the extent of damage of substances, animal experiments are more meaningful studies with regard to long-term effects. Plasma application was shown to lack genotoxic effects in vitro [30,32] and using an egg HET-MN model [31], which takes an intermediate position between cell culture and rodent models. Here, we have further quantitatively characterized the long-term side effects using an immunocompetent mouse model. In this model, inflammation and immune responses in wound repair are not attenuated, allowing us to follow not only wound healing in a physiological setting [45] but also the long-term impact on organs and skin tissue.

Cold plasmas are a significant source of reactive oxygen species (ROS, e.g., HO, O_2_^−^, O_3_, and H_2_O_2_) and reactive nitrogen species (RNS, e.g., NO, ONOO^−^, and NO_2_^−^) [46]. At low concentration, some of these reactive components are beneficial—leading to cell proliferation and survival pathways—and are considered second messengers in the field of redox biology. Nevertheless, these species can be detoxified and metabolically controlled in cells and organisms via anti-oxidant protection mechanisms. We have previously demonstrated in vitro that plasma significantly altered anti-oxidant and phase II detoxification enzymes and proteins such as glutathione peroxidases, catalase, superoxide dismutase, heme oxygenase 1, NAD(P)H dehydrogenase 1, mitogen-activated protein kinases, molecules of the Jun pathway, and growth factors to protect skin cells from oxidative stress [47,48,49,50]. At higher quantities, reactive species may be tumorigenic [51] and mutagenic [52]. Regardless of the plasma device or the treatment time, no genotoxic effects of the kINPen plasma were found using a HPRT1 mutation assay [30] or the HET-MN model [31]. In the latter study, the global antioxidant defense was also not significantly challenged in fertilized plasma-treated chicken eggs. Furthermore, a long exposure time of test agents is recommended to increase the possibility of tumor development and to receive resilient statements about tumorigenicity. The regular clinical protocol for plasma treatment of chronic wounds aims for two to three treatments per week and a treatment time of 30 to 60 s per cm^2^ [53]. Regarding these observations and preclinical plasma studies in humans [54,55,56,57], we decided on a daily treatment procedure of 20 s over 14 consecutive days, which seems advisable for toxicological investigations. Because of the brief exposure during the plasma treatment, acute health risks via ozone formation are not to be expected. The kINPen expels maximum ozone concentrations of 0.10–0.13 ppm at lower distances (<10 cm), which is in the range of the maximum working concentration value (MAK) of 0.1 ppm in the air [53].

Using whole body imaging as well as histological and biochemical analysis, our data suggest the absence of long-term side effects in the skin tissue and other organs of plasma-treated mice. This is in agreement with the literature where no histological damage was observed in human skin after 1 min [58] or 10 min of plasma treatment [59]. The absence of expression of a panel of tumor markers in liver, lung, brain, and ear tissue using qPCR suggests that plasma can support wound healing [45] without alterations in the tissue architecture and/or function. Additionally, serum levels were measured for AFP, a screening substance for a subset of abnormalities such as hepatocellular carcinoma [42] and calcitonin, which is produced in humans and rodents primarily by the parafollicular cells in the thyroid. A malignancy of thyroid glands (e.g., medullary thyroid carcinoma, C-cell hyperplasia, non-thyroidal carcinoma) typically produces elevated serum calcitonin levels of >5 ng/L in females, and 12 ng/L in males [60] and can therefore be used diagnostically as a tumor marker. Calcitonin and AFP serum levels were in the physiological range in plasma-treated and untreated animals of both genders.

Moreover, cervical and mesenteric lymph node and liver sections showed a strong inflammatory pathology in the HCC-bearing mouse as a response to a primary tumor formation in contrast to our plasma-treated animals. In the tumor-bearing mouse of the control group, alterations in the macrophage population indicated an activated state in the liver [36]. In all other animals in the plasma and control group, F4/80 positive cells within the liver confirmed the classic morphology of Kupffer cells consistent with an unaltered state of the liver. We also characterized the presence, morphology, and localization of granulocytes and macrophages in spleens suggesting that the mice exhibited dominant healthy features. Ly6G has also been implicated in the development of antitumor responses: It has been demonstrated that lymphoid cancers derive by chronic stimulation or dysfunction of B cells in association with a sustained inflammatory reaction [61]. Here, multisystem inflammatory reactions with cell infiltrations in several organs such as thyroid glands, spleen, and lymph nodes, or secretion of pro-inflammatory cytokines were not observed 350 days after plasma treatment.

Our study has limitations. First, we did not include positive controls in the animal experiments. Yet, in our view, it is not only experimentally too demanding but also unethical to include several dozens of different (possible) tumor groups with many animals each only for the sake of “generating” positive controls for downstream assays. Second, it was beyond the scope of this study to investigate every single tissue and organ in the animal body for possible side effects. Yet, using MRI and PET/CT scans, we believe to have sufficiently searched for any tumor formation in a large number of animals. Altogether, the findings of this study show the absence of long-term side effects after one year in mice receiving 14 consecutive plasma treatments. Hence, and given a profound efficacy, clinical plasma applications continue to be a promising therapy especially in dermatology.

## 4. Materials and Methods

### 4.1. Animals

A total of 84 SKH1-hr hairless immunocompetent mice (Charles River Laboratories, Sulzfeld, Germany) were housed under standard conditions in an animal facility (Rostock University Medical Center, Rostock, Germany). All experiments were approved by the local ethics committee according to the guidelines for care and use of laboratory animals (number 7221.3-1-013/14).

### 4.2. Wounding and Exposure to Cold Physical Plasma

Wounding was performed on the left and right ears of 10–12 weeks old animals as previously described [45]. Full-thickness circular dermal wounds of approximately 3 mm^2^ size were created by removing the epidermis and dermis but not the cartilage using a micro-scissor. For reproducibility, the procedure was carried out by a single operator. Mice were assigned randomly into four groups (*n* = 21, males and females), and either received daily treatment of plasma (20 s) or were left untreated over 14 consecutive days. The argon plasma jet *kINPen* 11 (neoplas tools, Greifswald, Germany) ionizes a flow (5 standard liters per minute) of argon gas at a frequency of 1 MHz [53]. Organs and tissues were collected 350 days post intervention.

### 4.3. Wound Closure Observations and Histological Evaluation

Wound tissues was fixed in 4% paraformaldehyde, and 5 µm-sections were stained with hematoxylin/eosin (H&E) to visualize morphological changes by means of light microscopy. The collagen content of lymph nodes was qualitatively determined within picrosirius red (PSR) stained paraffin-embedded tissue sections using fluorescence microscopy [38]. For immunohistostaining, paraffin sections were deparaffinized, rehydrated, and boiled for 5 min for antigen retrieval. Prior to imaging by light microscopy, sections were incubated with α-fetoprotein (MAB1368, R&D Systems, Germany), β-2-microglobulin (sc-15366, Santa Cruz, Heidelberg, Germany), neuron-specific enolase (#R30242, NSJ Bioreagents, Hamburg, Germany), and carcinoembryonic antigen (AF6480, R&D Systems, Wiesbaden, Germany) overnight at 4 °C. Detection of Kupffer cells and macrophages in paraffin-embedded liver and spleen sections was performed using anti-mouse F4/80 antibody (#14-4801; Affymetrix, Frankfurt, Germany). For the identification of monocytes and macrophages in the spleen, Ly6G was used (#14-5931; Affymetrix, Frankfurt, Germany). After washing, sections were incubated with an immuno-peroxidase polymer (N-Histofine staining reagent; Medac, Wedel, Germany). Stained sections were mounted onto glass microscope slides using mounting medium containing DAPI (VectaShield; Biozol, Eching, Germany) prior to analysis using an Axio Observer Z.1 (Zeiss, Jena, Germany).

### 4.4. Homogenisation of Tissues and Gene Expression Analysis

For expression analysis, tissue and organ samples were collected at day 350 (*n* = 84). Fresh tissues from ear and several organs (e.g., lung, liver, brain, thyroid, and lymph nodes) were snap-frozen in liquid nitrogen, and stored at −80 °C. For gene expression analysis, homogenization was performed in RNA lysis buffer (Bio&Sell, Nürnberg, Germany) using a FastPrep-24 5G homogenisator (Biomedicals, Heidelberg, Germany). Total RNA was isolated (Bio&Sell) and quantitative PCR (qPCR) was performed as described previously [49]. Briefly, 1 μg of RNA was transcribed into cDNA, and qPCR was conducted in triplicate using SYBR Green I Master in a 96-well LightCycler 480 qPCR system (Roche Diagnostics, Mannheim, Germany). Gene primers were used from Biomol (Hamburg, Germany) and Biotez (Berlin, Germany; Table A1). The housekeeping gene *GAPDH* expression was unaffected by plasma and was used as an internal control for normalization. Genes expression was analyzed using the ΔΔ*C*t method [62]. The final value for gene expression was determined by calculating the ratio of expression in the respective sample related to the control. Band intensities were quantified using ImageQuantTL Software (GE Healthcare, Freiburg, Germany), and expressed as fold change compared to the corresponding control. Experiments were performed with six to ten mice for each group.

### 4.5. ELISA Measurements and Bead-Based Cytokine Analysis

Blood serum were collected retrobulbar in EDTA-tubes (d0, d15 or d350), and centrifuged. Samples were stored until use at −80 °C. TNFα and IL-1β secretion was measured using bead-based multiplex cytokine analysis (BioLegend,Uithoorn, The Netherlands). Enzyme-linked immunosorbent assay (ELISA) for a liver specific tumor marker “α fetoprotein” (AFP, BioLegend, Uithoorn, The Netherlands) and a medullary thyroid carcinoma specific tumor marker “calcitonin” (CT, BioLegend, Uithoorn, The Netherlands) was performed.

### 4.6. MRI, PET/CT Scans, and Interpretation of Data

For positron emission tomography-computed tomography (PET/CT) and magnetic resonance imaging (MRI) sessions 350 days post intervention, animals were anaesthetized with isoflurane (1.5–2.5%) supplemented with oxygen. During both scans, respiration of the mice was monitored and adjusted to a breathing rate of 35–50 breaths/min. All MRI scans were additionally respiration triggered. Temperature of mice were also controlled during the entire imaging period of all scans. Anatomical MRI T2-weighted TurboRARE sequences with coronal and axial slices over the whole body were acquired with the following parameters: TE/TR: 29/4100 ms, FoV: cor. 94.4 mm × 35 mm, axial 33.7 mm × 37 mm, in-plane resolution 0.18 mm, slice thickness 1 mm. For small animal PET/CT imaging, mice were injected intravenously with 15 MBq ^18^F-FDG via a microcatheter placed in the tail vein. Static PET scans in prone position (duration: 15 min) were performed starting 60 min after tracer application using the Inveon dedicated PET/CT scanner (Siemens Preclinical Solutions, Knoxville, TN, USA). Whole body CT scans were acquired for attenuation correction and anatomical reference. CT images were reconstructed with a Feldkamp algorithm. PET data were first Fourier rebinned into a 2D dataset from which real-space images were reconstructed with an ordered subset expectation maximization (OSEM) algorithm with 16 subsets and 4 iterations. Attenuation correction was carried out using the CT data.

### 4.7. Statistical Analysis

Means of at least three independent experiments were calculated using *prism* software (GraphPad, La Jolla, CA, USA). Data were subjected to statistical analysis using the unpaired Student’s *t* test or one-way analysis of variance (ANOVA) followed by the Dunnett’s post hoc test. Significant differences were indicated as mean + S.D. with *p*-values from * *p* < 0.05, ** *p* < 0.01, *** *p* < 0.001.

## 5. Conclusions

One year after fourteen consecutive treatments of murine full-thickness ear wounds with the argon plasma jet kINPen, we were able to conclude key aspects with regard to efficacy and safety of that device. First, skin tissue healed physiologically without excessive scar formation similar to control animals. Second, the application of plasma was safe as demonstrated by the absence of tumor formation at the treatment site. Third, no malignant tissue was found in any other major organ including liver, lymph node, spleen, heart, lung, and brain. Fourth, the lack of neoplastic markers in blood plasma of plasma-treated animals suggested an absence of tumor tissue at sites in the murine body not investigated in this study. Fifth, the analysis of cytokines and immune cells indicated a physiological immune regulation without pathological or excessive inflammation. These data suggest that cold plasma applied topically is a safe adjuvant strategy in dermatology with regard to the absence of neoplastic side effects.

## Abbreviations

FoV	field of view
MRI	magnetic resonance imaging
PET/CT	positron emission tomography/computed tomography
ROS	reactive oxygen species
RNS	reactive nitrogen species
TE	echo time
TR	repetition time

## Appendix A

**Table A1:** Murine primer sequences or catalog numbers (Biomol, Germany) for use in quantitative real-time PCR.

Name	Gene	Sequences/# (Biomol)
*mGAPDH_s*	House keeping gene	CATGGCCTCCAAGGAGTAAG
*mGAPDH_as*		TGTGAGGGAGATGCTCAGTG
*mTNFα_s*	Tumor necrosis factor α	TCTCATGCACCACCATCAAGGACT
*mTNFα_as*		ACCACTCTCCCTTTGCAGAACTCA
*mIL-1β_s*	Interleukin 1β	GCAACTGTTCCTGAACTCAACT
*mIL-1β_as*		ATCTTTTGGGGTCCGTCAACT
*mAFP_s*	α fetoprotein	CCAGGAAGTCTGTTTCACAGAAG
*mAFP_as*		CAAAAGGCTCACACCAAAGAG
*mβ2M*	β2 microglobulin	VMPS-563
*mCEA1*	Carcinoembryonic antigen	VMPS-1054
*mCT*	Calcitonin	VMPS-785
*mNSE*	Neuron specific enolase	VMPS-1942
